# Clinical significance of putative markers of cancer stem cells in gastric cancer: A retrospective cohort study

**DOI:** 10.18632/oncotarget.11384

**Published:** 2016-08-19

**Authors:** Xiao-Long Chen, Xin-Zu Chen, Yi-Gao Wang, Du He, Zheng-Hao Lu, Kai Liu, Wei-Han Zhang, Wei Wang, Chang-Chun Li, Lian Xue, Lin-Yong Zhao, Kun Yang, Jian-Ping Liu, Zong-Guang Zhou, Jian-Kun Hu, Xian-Ming Mo

**Affiliations:** ^1^ Department of Gastrointestinal Surgery, West China Hospital, Sichuan University, Chengdu 610041, China; ^2^ Laboratory of Gastric Cancer, State Key Laboratory of Biotherapy/Collaborative Innovation Center of Biotherapy and Cancer Center, West China Hospital, Sichuan University, Chengdu 610041, China; ^3^ Department of Pathology, West China Hospital, Sichuan University, Chengdu 610041, China; ^4^ Institute of Digestive Surgery, State Key Laboratory of Biotherapy/Collaborative Innovation Center of Biotherapy and Cancer Center, West China Hospital, Sichuan University, Chengdu 610041, China; ^5^ Laboratory of Stem Cell Biology, State Key Laboratory of Biotherapy/Collaborative Innovation Center of Biotherapy and Cancer Center, West China Hospital, Sichuan University, Chengdu 610041, China

**Keywords:** gastric cancer, cancer stem cells, markers, prognosis, nomogram

## Abstract

Cancer stem cells (CSCs) are thought as the source of tumor maintaining and many CSCs markers have been identified. Regarding the heterogeneity in gastric cancer (GC), TNM stage is not enough to accurately predict the prognosis. The aim of this study was to investigate the clinical significance of CSCs markers (Lgr5, Oct4, CD133, EpCAM, CD54 and Sox2) and establish a new model based on these markers to accurately predict prognosis of GC. We retrospectively enrolled 377 GC tissues from January 2006 to October 2012 to perform immunohistochemistry (IHC), and 93 pairs of GC tissues and corresponding adjacent normal gastric tissues to perform quantitative PCR (qPCR) from December 2011 to October 2012. The clinicopathological and follow-up characteristics were collected. In IHC, Oct4, CD133 and EpCAM were independently related to tumor progression, while Sox2 were associated with well or moderate differentiation (all p<0.05). Cox regression showed that Oct4-EpCAM was an independently prognostic factor, indicating that double low expression of Oct4-EpCAM group had significantly better prognosis than control group (p=0.035). Regarding qPCR, CD133 was an independent prognostic factor, showing that the prognosis of patients with CD133 high expression was significantly worse than that of patients with CD133 low expression (p<0.001). The prognostic prediction accuracy of nomogram based on Oct4-EpCAM expression in IHC was significantly better than TNM stage alone (p=0.003). Low expressions of Oct4-EpCAM in IHC and CD133 in qPCR were favorable prognostic factors in GC. The nomogram based on Oct4-EpCAM was valuable in prognostic prediction of GC patients.

## INTRODUCTION

Gastric cancer (GC) is one of the leading causes of cancer-related mortality worldwide, with high incidence in Asia [1]. For patients with resectable GC, surgery and adjuvant chemoradiotherapy are the main way to cure this malignance. However, many patients still suffered recurrence and metastasis, though they received standard treatments. The concept of cancer stem cells (CSCs) has been put forward to explain the cause of therapy resistance and many studies have discovered that CSCs might play a pivotal role in tumor recurrence and metastasis [2, 3]. Meanwhile, some specific markers or their combinations have also been demonstrated valuable in identifying CSCs. These specific markers might be the key points in target therapy and prognostic prediction. From previous studies, we found that intercellular adhesion molecule 1 (CD54), leucine rich repeat containing G protein coupled receptor 5 (Lgr5), prominin 1 (CD133), POU class 5 homeobox 1 (Oct4), epithelial cell adhesion molecule (EpCAM) and sex determining region Y-box 2 (Sox2) were demonstrated as the putative markers of CSCs in many kinds of tumors [4–6]. The relationship between these markers and clinicopathological characteristics and the prognostic significance of these markers have been investigated in GC [7, 8]. However, many studies only focused on several of these six markers and the results were still controversial. Therefore, the significance of these markers were still under debate and should be further demonstrated.

Nowadays, TNM stage revealing tumor invasion depth, regional metastatic lymph nodes (LNs) and distant metastasis is one of the most important classifications of tumor progression and a useful clinical tool in prognostic prediction of GC [1, 9]. Nevertheless, TNM stage cannot illustrate complete information of tumors and patients. As far as we know, heterogeneity extensively exists in many tumors [10, 11]. In clinical practice, some patients with the same TNM stage are found to have different prognosis. Therefore, it is necessary to find new tools or crucial supplementary of TNM stage that represents the individual characteristics to accurately predict the prognosis of patients with GC. The aim of this study was to investigate the clinical significance of these six markers of CSCs and establish a new model based on these markers to predict the prognosis of patients with GC.

## RESULTS

### Expressions of these markers in gastric CSCs (GCSCs) and primary lesions

**Figure 1:**
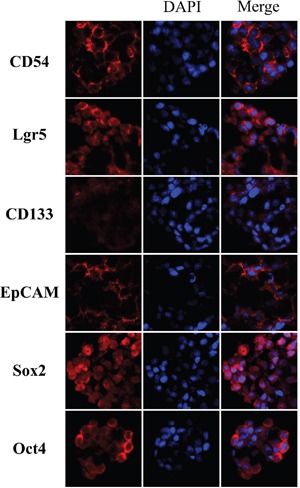
Immunofluorescence of gastric cancer stem cells spheres

**Figure 2:**
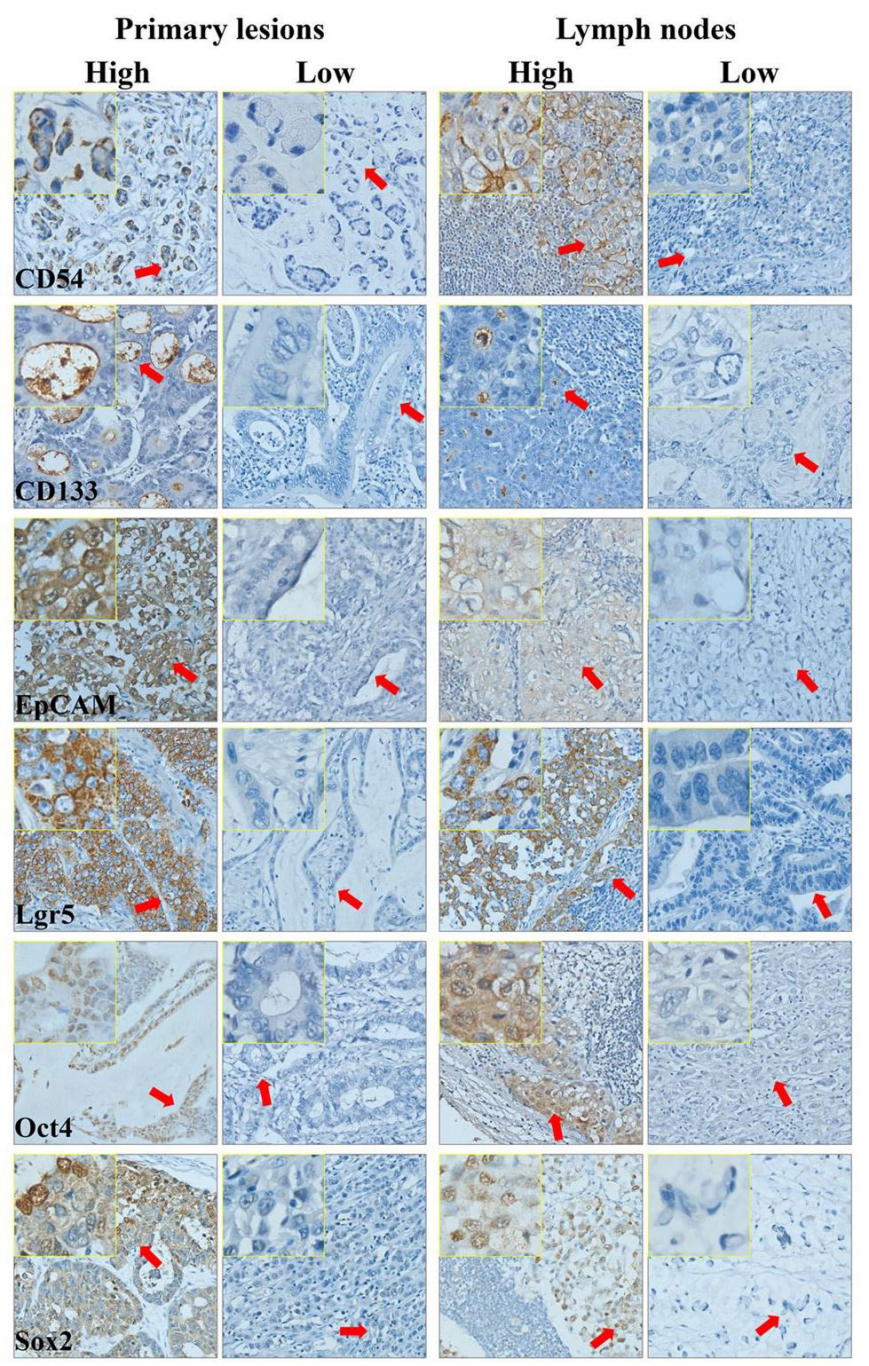
Immunohistochemistry of primary lesions and metastatic lymph nodes of gastric cancer

In GCSCs, EpCAM and CD54 were highly expressed on the cytomembrane, CD133 was weakly but Lgr5 and Oct4 were highly expressed in cytoplasm, and Sox2 was highly expressed in cytoplasm and nucleus (Figure 1). The expressions of these markers in primary lesions and metastatic LNs of GC through IHC were similar to GCSCs, except that CD133 was mainly expressed in the lumen of glands and Oct4 was expressed in nucleus (Figure 2). The relationships among these six markers in primary lesions were also analyzed and showed that EpCAM had significantly negative correlation to Lgr5 (p=0.007), CD133 (p=0.006) and Oct4 (p=0.001), while Oct4 was positively associated with Lgr5 (p<0.001) and CD133 (p=0.024) in immunohistochemistry (IHC). With respect to quantitative polymerase chain reaction (qPCR), we found that Lgr5 and Sox2 were remarkably positively correlated with CD54 (p=0.006) and Oct4 (p=0.006), respectively (Table 1).

**Table 1:** Relationship among these six markers in primary lesions in IHC and qPCR

	CD54	Lgr5	CD133	Oct4	EpCAM	Sox2
CD54		*0.006 (P)*	*0.078*	*0.698*	*0.634*	*0.530*
Lgr5	**0.050**		*0.366*	*0.329*	*0.668*	*0.641*
CD133	**0.403**	**0.104**		*0.264*	*0.359*	*0.602*
Oct4	**0.877**	**<0.001 (P)**	**0.024 (P)**		*0.141*	*0.006 (P)*
EpCAM	**0.593**	**0.007 (N)**	**0.006 (N)**	**0.001 (N)**		*0.981*
Sox2	**0.997**	**0.186**	**0.106**	**0.123**	**0.619**	

Note: Bold numbers represent p values of IHC and italic numbers represent p values of qPCR. P: positive correlation; N: negative correlation.

### Relationship between the expressions of these markers in primary lesions and clinicopathological characteristics

Univariate correlated analyses of IHC and qPCR were respectively shown in Table 2 and Table 3. Multivariate analyses of IHC were shown in Table 4.

**Table 2:** Clinicopathological features of patients tested by immunohistochemistry in this study

Clinicopathological Features	CD54			Lgr5			CD133			Oct4			EpCAM			Sox2		
	Low	High		Low	High		Low	High		Low	High		Low	High		Low	High	
	n=321	n=56	p value	n=276	n=101	p value	n=311	n=66	p value	n=266	n=111	p value	n=123	n=254	p value	n=211	n=96	p value
	(%)	(%)		(%)	(%)		(%)	(%)		(%)	(%)		(%)	(%)		(%)	(%)	
Age (years)																		
Mean	56.6	59.6	0.082	55.8	60.5	0.001	56.1	61.7	<0.001	56.4	58.7	0.074	53.9	58.6	<0.001	56.4	57.2	0.570
SD	11.6	12.4		11.7	11.2		11.8	10.3		11.6	12.0		11.0	11.8		12.1	11.0	
≤45	66 (21)	8 (14)	0.066	64 (23)	10 (10)	0.001	70 (23)	4 (6)	0.001	56 (21)	18 (16)	0.020	34 (28)	40 (16)	<0.001	45 (21)	18 (19)	0.655
45-60	131 (41)	19 (34)		112 (41)	38 (38)		124 (40)	26 (39)		113 (42)	37 (33)		54 (44)	96 (38)		84 (40)	39 (41)	
>60	124 (39)	29 (52)		100 (36)	53 (52)		117 (38)	36 (55)		97 (36)	56 (50)		35 (28)	118 (46)		82 (39)	39 (41)	
Gender			0.299			0.024			0.079			0.020			0.447			0.139
Male	237 (74)	45 (80)		198 (72)	84 (83)		227 (73)	55 (83)		190 (71)	92 (83)		89 (72)	193 (76)		155 (73)	78 (81)	
Female	84 (26)	11 (20)		78 (28)	17 (17)		84 (27)	11 (17)		76 (29)	19 (17)		34 (28)	61 (24)		56 (27)	18 (19)	
Longitudinal location			0.285			<0.001			0.003			0.059			0.040			0.330
U	79 (25)	19 (34)		64 (23)	34 (34)		69 (22)	29 (44)		70 (26)	28 (25)		21 (17)	77 (30)		62 (29)	23 (24)	
M	78 (24)	10 (18)		79 (29)	9 (9)		77 (25)	11 (17)		71 (27)	17 (15)		33 (27)	55 (22)		48 (23)	19 (20)	
L	158 (49)	25 (45)		128 (46)	55 (54)		159 (51)	24 (36)		119 (45)	64 (58)		66 (54)	117 (46)		94 (45)	53 (55)	
UML	6 (2)	2 (4)		5 (2)	3 (3)		6 (2)	2 (3)		6 (2)	2 (2)		3 (2)	5 (2)		7 (3)	1 (1)	
Cross sectional location			0.974			0.787			0.365			0.418			0.252			0.111
Lesser	162 (50)	29 (52)		142 (51)	49 (49)		156 (50)	35 (53)		142 (53)	49 (44)		56 (46)	135 (53)		102 (48)	54 (56)	
Greater	29 (9)	6 (11)		24 (9)	11 (11)		33 (11)	2 (3)		21 (8)	14 (13)		11 (9)	24 (9)		23 (11)	9 (9)	
Anterior	16 (5)	3 (5)		15 (5)	4 (4)		16 (5)	3 (5)		14 (5)	5 (5)		10 (8)	9 (4)		10 (5)	3 (3)	
Posterior	24 (7)	4 (7)		22 (8)	6 (6)		23 (7)	5 (8)		19 (7)	9 (8)		8 (7)	20 (8)		21 (10)	2 (2)	
Multiple	90 (28)	14 (25)		73 (26)	31 (31)		83 (27)	21 (32)		70 (26)	34 (31)		38 (31)	66 (26)		55 (26)	28 (29)	
Macroscopic type			0.129			0.030			0.047			<0.001			0.020			0.225
0	41 (13)	3 (5)		35 (13)	9 (9)		42 (14)	2 (3)		38 (14)	6 (5)		24 (20)	20 (8)		28 (13)	11 (11)	
I	10 (3)	0 (0)		9 (3)	1 (1)		9 (3)	1 (2)		10 (4)	0 (0)		5 (4)	5 (2)		2 (1)	4 (4)	
II	148 (46)	28 (50)		135 (49)	41 (41)		143 (46)	33 (50)		128 (48)	48 (43)		52 (42)	124 (49)		85 (40)	45 (47)	
III	92 (29)	19 (34)		69 (25)	42 (42)		89 (29)	22 (33)		68 (26)	43 (39)		30 (24)	81 (32)		72 (34)	29 (30)	
IV	30 (9)	6 (11)		28 (10)	8 (8)		28 (9)	8 (12)		22 (8)	14 (13)		12 (10)	24 (9)		24 (11)	7 (7)	
Differentiation			0.285			0.096			0.014			0.331			0.685			0.042
Well/Moderately	36 (11)	3 (5)		22 (8)	17 (17)		27 (9)	12 (18)		27 (10)	12 (11)		12 (10)	27 (11)		19 (9)	15 (16)	
Moderately-poorly	52 (16)	9 (16)		46 (17)	15 (15)		48 (15)	13 (20)		48 (18)	13 (12)		19 (15)	42 (17)		25 (12)	15 (16)	
Poorly	233 (73)	44 (79)		208 (75)	69 (68)		236 (76)	41 (62)		191 (72)	86 (77)		92 (75)	185 (73)		167 (79)	66 (69)	
Tumor size (cm)																		
Mean±SD	5.8±3.1	6.3±2.8	0.215	5.7±3.2	6.2±2.8	0.183	5.7±3.1	6.4±3.0	0.086	5.6±3.0	6.5±3.2	0.010	5.1±2.9	6.2±3.1	0.001	6.1±3.2	5.9±3.3	0.598
≤4	119 (37)	14 (25)	0.172	108 (39)	25 (25)	0.044	117 (38)	16 (24)	0.099	100 (38)	33 (30)	0.023	56 (46)	77 (30)	0.002	65 (31)	38 (40)	0.489
4-7	124 (39)	27 (48)		102 (37)	49 (49)		119 (38)	32 (48)		110 (41)	41 (37)		46 (37)	105 (41)		90 (43)	30 (31)	
>7	78 (24)	15 (27)		66 (24)	27 (27)		75 (24)	18 (27)		56 (21)	37 (33)		21 (17)	72 (28)		56 (27)	28 (29)	
Vessels/nerves invasion			0.344			0.050			0.651			0.071			0.599			0.364
Negative	248 (77)	40 (71)		218 (79)	70 (69)		239 (77)	49 (74)		210 (79)	78 (70)		96 (78)	192 (76)		168 (80)	72 (75)	
Positive	73 (23)	16 (29)		58 (21)	31 (31)		72 (23)	17 (26)		56 (21)	33 (30)		27 (22)	62 (24)		43 (20)	24 (25)	
T stage			0.047			0.432			0.095			0.056			0.017			0.616
1	45 (14)	4 (7)		37 (13)	12 (12)		47 (15)	2 (3)		42 (16)	7 (6)		26 (21)	23 (9)		31 (15)	12 (13)	
2	27 (8)	4 (7)		27 (10)	4 (4)		26 (8)	5 (8)		22 (8)	9 (8)		13 (11)	18 (7)		15 (7)	3 (3)	
3	22 (7)	1 (2)		14 (5)	9 (9)		16 (5)	7 (11)		15 (6)	8 (7)		2 (2)	21 (8)		13 (6)	10 (10)	
4	227 (71)	47 (84)		198 (72)	76 (75)		222 (71)	52 (79)		187 (70)	87 (78)		82 (67)	192 (76)		152 (72)	71 (74)	
N stage			0.085			0.143			0.016			0.003			0.019			0.491
0	90 (28)	12 (21)		83 (30)	19 (19)		95 (31)	7 (11)		80 (30)	22 (20)		45 (37)	57 (22)		58 (27)	26 (27)	
1	48 (15)	3 (5)		37 (13)	14 (14)		43 (14)	8 (12)		41 (15)	10 (9)		15 (12)	36 (14)		22 (10)	15 (16)	
2	59 (18)	14 (25)		47 (17)	26 (26)		51 (16)	22 (33)		50 (19)	23 (21)		20 (16)	53 (21)		38 (18)	18 (19)	
3	124 (39)	27 (48)		109 (39)	42 (42)		122 (39)	29 (44)		95 (36)	56 (50)		43 (35)	108 (43)		93 (44)	37 (39)	
M stage			0.459			0.965			0.865			0.011			0.748			0.767
0	281 (88)	47 (84)		240 (87)	88 (87)		271 (87)	57 (86)		239 (90)	89 (80)		108 (88)	220 (87)		182 (86)	84 (88)	
1	40 (12)	9 (16)		36 (13)	13 (13)		40 (13)	9 (14)		27 (10)	22 (20)		15 (12)	34 (13)		29 (14)	12 (13)	
TNM stage			0.094			0.046			0.018			0.001			0.004			0.934
I	54 (17)	7 (13)		50 (18)	11 (11)		60 (19)	1 (2)		50 (19)	11 (10)		31 (25)	30 (12)		38 (18)	12 (13)	
II	55 (17)	5 (9)		49 (18)	11 (11)		48 (15)	12 (18)		46 (17)	14 (13)		22 (18)	38 (15)		27 (13)	18 (19)	
III	172 (54)	35 (63)		141 (51)	66 (65)		163 (52)	44 (67)		143 (54)	64 (58)		55 (45)	152 (60)		117 (55)	54 (56)	
IV	40 (12)	9 (16)		36 (13)	13 (13)		40 (13)	9 (14)		27 (10)	22 (20)		15 (12)	34 (13)		29 (14)	12 (13)	
Chemotherapy			0.980			0.149			0.745			0.553			0.003			0.595
No	184 (57)	32 (57)		152 (55)	64 (63)		177 (57)	39 (59)		155 (58)	61 (55)		57 (46)	159 (63)		132 (63)	57 (59)	
Yes	137 (43)	24 (43)		124 (45)	37 (37)		134 (43)	27 (41)		111 (42)	50 (45)		66 (54)	95 (37)		79 (37)	39 (41)	

**Table 3:** Clinicopathological features of patients tested by quantitative PCR in this study

Clinicopathological Features	CD54			Lgr5			CD133			Oct4			EpCAM			Sox2		
	Low	High		Low	High		Low	High		Low	High		Low	High		Low	High	
	n=53	n=40	p value	n=50	n=43	p value	n=69	n=24	p value	n=44	n=49	p value	n=65	n=28	p value	n=43	n=50	p value
	(%)	(%)		(%)	(%)		(%)	(%)		(%)	(%)		(%)	(%)		(%)	(%)	
Age (years)																		
Mean	59.4	61.1		58.6	62.0		61.2	57.1		61.6	58.9		60.0	60.5		63.6	57.2	
SD	12.7	10.6	0.498	11.9	11.5	0.160	11.8	11.7	0.140	10.5	12.9	0.287	12.0	11.6	0.857	9.5	12.9	0.010
≤60	26 (49)	18 (45)	0.698	25 (50)	19 (44)	0.576	31 (45)	13 (54)	0.435	18 (41)	26 (53)	0.241	32 (49)	12 (43)	0.572	14 (33)	30 (60)	0.008
>60	27 (51)	22 (55)		25 (50)	24 (56)		38 (55)	11 (46)		26 (59)	23 (47)		33 (51)	16 (57)		29 (67)	20 (40)	
Gender			0.543			0.253			1.000			0.3			0.335			0.357
Male	41 (77)	33 (83)		42 (84)	32 (74)		55 (80)	19 (79)		33 (75)	41 (84)		50 (77)	24 (86)		36 (84)	38 (76)	
Female	12 (23)	7 (18)		8 (16)	11 (26)		14 (20)	5 (21)		11 (25)	8 (16)		15 (23)	4 (14)		7 (16)	12 (24)	
Longitudinal location			0.672			0.984			0.250			0.984			0.657			0.095
U	19 (36)	18 (45)		19 (38)	18 (42)		29 (42)	8 (33)		17 (39)	20 (41)		26 (40)	11 (39)		14 (33)	23 (46)	
M	7 (13)	7 (18)		8 (16)	6 (14)		9 (13)	5 (21)		7 (16)	7 (14)		8 (12)	6 (21)		6 (14)	8 (16)	
L	25 (47)	14 (35)		21 (42)	18 (42)		30 (43)	9 (38)		19 (43)	20 (41)		29 (45)	10 (36)		23 (53)	16 (32)	
UML	2 (4)	1 (3)		2 (4)	1 (2)		1 (1)	2 (8)		1 (2)	2 (4)		2 (3)	1 (4)		0 (0)	3 (6)	
Cross sectional location			0.082			0.280			0.886			0.412			0.157			0.933
Lesser	25 (47)	14 (35)		21 (42)	18 (42)		27 (39)	12 (50)		17 (39)	22 (45)		22 (34)	17 (61)		17 (40)	22 (44)	
Greater	3 (6)	7 (18)		4 (8)	6 (14)		8 (12)	2 (8)		4 (9)	6 (12)		9 (14)	1 (4)		4 (9)	6 (12)	
Anterior	3 (6)	0 (0)		1 (2)	2 (5)		3 (4)	0 (0)		0 (0)	3 (6)		3 (5)	0 (0)		2 (5)	1 (2)	
Posterior	8 (15)	3 (8)		9 (18)	2 (5)		8 (12)	3 (13)		6 (14)	5 (10)		9 (14)	2 (7)		5 (12)	6 (12)	
Multiple	14 (26)	16 (40)		15 (30)	15 (35)		23 (33)	17 (71)		17 (39)	13 (27)		22 (34)	8 (29)		15 (35)	15 (30)	
Macroscopic type			0.569			0.547			0.567			0.681			0.104			0.149
I-II	23 (43)	15 (38)		19 (38)	19 (44)		27 (39)	11 (46)		17 (39)	21 (43)		23 (35)	15 (54)		21 (49)	17 (34)	
III-IV	30 (57)	25 (63)		31 (62)	24 (56)		42 (61)	13 (54)		27 (61)	28 (57)		42 (65)	13 (46)		22 (51)	33 (66)	
Differentiation			0.569			0.067			0.052			0.335			0.174			0.513
Well/Moderately	6 (11)	7 (18)		3 (6)	10 (23)		12 (17)	1 (4)		7 (16)	6 (12)		8 (12)	5 (18)		7 (16)	6 (12)	
Moderately-poorly	5 (9)	3 (8)		5 (10)	3 (7)		7 (10)	1 (4)		5 (11)	3 (6)		4 (6)	4 (14)		4 (9)	4 (8)	
Poorly	42 (79)	30 (75)		42 (84)	30 (70)		50 (72)	22 (92)		32 (73)	40 (82)		53 (82)	19 (68)		32 (74)	40 (80)	
Tumor size (cm)																		
Mean±SD	7.4±2.5	7.1±2.8	0.630	7.3±2.7	7.3±2.5	0.989	7.3±2.6	7.1±2.8	0.730	7.3±2.5	7.2±2.7	0.911	7.6±2.8	6.4±1.8	0.085	6.9±1.9	7.6±3.1	0.510
≤7	31 (58)	27 (68)	0.344	32 (64)	26 (60)	0.879	42 (61)	16 (67)	0.614	26 (59)	32 (65)	0.898	37 (57)	21 (75)	0.099	28 (65)	30 (60)	0.614
>7	22 (42)	13 (33)		18 (36)	17 (40)		27 (39)	8 (33)		18 (41)	17 (35)		28 (43)	7 (25)		15 (35)	20 (40)	
Vessels/nerves invasion			0.907			0.255			0.440			0.394			0.810			0.794
Negative	39 (74)	29 (73)		39 (78)	29 (67)		49 (71)	19 (79)		34 (77)	34 (69)		48 (74)	20 (71)		32 (74)	36 (72)	
Positive	14 (26)	11 (28)		11 (22)	14 (33)		20 (29)	5 (21)		10 (23)	15 (31)		17 (26)	8 (29)		11 (26)	14 (28)	
T stage			0.993			0.549			0.107			0.035			0.072			0.030
1-2	4 (8)	3 (8)		3 (6)	4 (9)		7 (10)	0 (0)		6 (14)	1 (2)		7 (11)	0 (0)		6 (14)	1 (2)	
3-4	49 (92)	37 (93)		47 (94)	39 (91)		62 (90)	24 (100)		38 (86)	48 (98)		58 (89)	28 (100)		37 (86)	49 (98)	
N stage			0.760			0.405			0.282			0.657			0.900			0.726
0	5 (9)	5 (13)		5 (10)	5 (12)		7 (10)	3 (13)		4 (9)	6 (12)		6 (9)	4 (14)		5 (12)	5 (10)	
1	6 (11)	5 (13)		5 (10)	6 (14)		10 (14)	1 (4)		4 (9)	7 (14)		10 (15)	1 (4)		7 (16)	4 (8)	
2	12 (23)	8 (20)		10 (20)	10 (23)		16 (23)	4 (17)		11 (25)	9 (18)		12 (18)	8 (29)		7 (16)	13 (26)	
3	30 (57)	22 (55)		30 (60)	22 (51)		36 (52)	16 (67)		25 (57)	27 (55)		37 (57)	15 (54)		24 (56)	28 (56)	
M stage			0.380			0.971			0.934			0.285			0.006			0.276
0	46 (87)	32 (80)		42 (84)	36 (84)		58 (84)	20 (83)		35 (80)	43 (88)		50 (77)	28 (100)		38 (88)	40 (80)	
1	7 (13)	8 (20)		8 (16)	7 (16)		11 (16)	4 (17)		9 (20)	6 (12)		15 (23)	0 (0)		5 (12)	10 (20)	
TNM stage			0.249			0.377			0.288			0.828			0.445			0.761
I-II	6 (11)	8 (20)		6 (12)	8 (19)		12 (17)	2 (8)		7 (16)	7 (14)		11 (17)	3 (11)		7 (16)	7 (14)	
III-IV	47 (89)	32 (80)		44 (88)	35 (81)		57 (83)	22 (92)		37 (84)	42 (86)		54 (83)	25 (89)		36 (84)	43 (86)	
Chemotherapy			0.835			0.713			0.963			0.164			0.981			0.105
No	38 (53)	22 (55)		26 (52)	24 (56)		37 (54)	13 (54)		27 (61)	23 (47)		35 (54)	15 (54)		27 (63)	23 (46)	
Yes	25 (47)	18 (45)		24 (48)	19 (44)		32 (46)	11 (46)		17 (39)	26 (53)		30 (46)	13 (46)		16 (37)	27 (54)	

**Table 4:** Multivariate logistic regression of the patients tested by immunohistochemistry

Clinicopathological Features	CD54(n=377)			Lgr5(n=377)			CD133(n=377)			Oct4(n=377)			EpCAM (n=377)			Sox2 (n=307)		
	p value	OR	95%CI	p value	OR	95%CI	p value	OR	95%CI	p value	OR	95%CI	p value	OR	95%CI	p value	OR	95%CI
Age	—	—	—	0.001	1.756	1.263-2.442	0.008	1.769	1.162-2.692	—	—	—	0.001	1.619	1.204-2.178	—	—	—
Gender	—	—	—	—	—	—	—	—	—	0.014	0.478	0.275-0.864	—	—	—	—	—	—
Longitudinal location	—	—	—	—	—	—	0.002	0.694	0.509-0.948	—	—	—	—	—	—	—	—	—
Cross sectional location	—	—	—	—	—	—	—	—	—	—	—	—	—	—	—	—	—	—
Macroscopic type	—	—	—	—	—	—	—	—	—	<0.001	1.559	1.228-1.979	—	—	—	—	—	—
Differentiation	—	—	—	—	—	—	0.013	0.600	0.401-0.900	—	—	—	—	—	—	0.041	0.699	0.495-0.986
Tumor size	—	—	—	—	—	—	—	—	—	—	—	—	0.008	1.492	1.109-2.008	—	—	—
Vessels/nerves invasion	—	—	—	—	—	—	—	—	—	—	—	—	—	—	—	—	—	—
T stage	—	—	—	—	—	—	—	—	—	—	—	—	—	—	—	—	—	—
N stage	—	—	—	—	—	—	0.003	1.504	1.154-1.961	—	—	—	—	—	—	—	—	—
M stage	—	—	—	—	—	—	—	—	—	—	—	—	—	—	—	—	—	—

### Lgr5

In IHC, 276 (73.2%) and 101 (26.8%) patients were in Lgr5 low and Lgr5 high expression groups, respectively. The results showed that Lgr5 high expression group had remarkably more patients with >60 years (p=0.001), male (p=0.024), macroscopic type III (p=0.030), tumor size 4cm-7cm (p=0.044) and TNM III stage (p=0.046) than Lgr5 low expression group. Multivariate analysis revealed that Lgr5 expression was only independently associated with age (p=0.001, OR=1.756, 95%CI [1.263-2.442]).

In qPCR, there were 50 (53.8%) patients in Lgr5 low expression group and 43 (46.2%) patients in Lgr5 high expression group. We found that well or moderate differentiation grade was significantly independently related to Lgr5 high expression (p=0.041, OR=0.527, 95%CI [0.286-0.973]).

### Oct4

In IHC, the patients were divided into Oct4 low (n=266, 70.6%) and high (n=111, 29.4%) expression groups. Oct4 high expression was significantly related to age >60 years (p=0.020), male (p=0.020), macroscopic type III-IV (p<0.001), tumor size >7cm (p=0.023), N3 stage (p=0.003), M1 stage (p=0.011) and TNM III-IV stage (p=0.001). Multivariate analysis showed that gender (p=0.014, OR=0.487, 95%CI [0.275-0.864]) and macroscopic type (p<0.001, OR=1.559, 95%CI [1.228-1.979]) were independent related factors to Oct4 expression.

In qPCR, 44 (47.3%) and 49 (52.7%) patients were divided into Oct4 low and Oct4 high expression groups, respectively. We found that T stage was significantly correlated with Oct4 expression (p=0.035), however, no independent related clinicopathological characteristics to Oct4 were found.

### CD133

In IHC, there were 311 (82.5%) and 66 (17.5%) patients in CD133 low and high expression groups, respectively. CD133 high expression had obviously relation to age >60 years (p=0.001), upper parts of stomach (p=0.003), macroscopic type IV (p=0.047), well or moderate differentiation grade (p=0.014), N2 stage (p=0.016) and TNM III stage (p=0.018). In multivariate analysis, age (p=0.008, OR=1.769, 95%CI [1.162-2.692]), longitudinal location (p=0.022, OR=0.694, 95%CI [0.509-0.948]), differentiation grade (p=0.014, OR=0.600, 95%CI [0.401-0.900]) and N stage (p=0.003, OR=1.504, 95%CI [1.154-1.961]) were independently related factors.

In qPCR, there were 69 (74.2%) in CD133 low expression group and 24 (25.8%) patients in CD133 high group. But no significantly related factors to CD133 expression were found.

### EpCAM

In IHC, the patients were divided into EpCAM low (n=123, 32.6%) and high (n=254, 67.4%) expression groups. EpCAM high expression significantly had something to do with to age >60 years (p<0.001), upper parts of stomach (p=0.040), macroscopic type III (p=0.020), tumor size >7cm (p=0.002), T3 stage (p=0.017), N2-3 stage (p=0.019) and TNM III stage (p=0.004). Multivariate analysis showed that age (p=0.001, OR=1.619, 95%CI [1.204-2.178]) and tumor size (p=0.008, OR=1.492, 95%CI [1.109-2.008]) were independent related factors to EpCAM expression.

In qPCR, there were 65 (69.9%) and 28 (30.1%) patients in EpCAM low and high expression groups. EpCAM expression was remarkably concerned in M stage (p=0.006) in univariate analysis, but no clinicopathological traits were significantly related to EpCAM expression in multivariate analysis.

### CD54

In IHC, there were 321 (85.1%) patients in CD54 low expression and 56 (14.9%) patients in CD54 high expression group. CD54 expression was only significantly related to T stage (p=0.047) in univariate analysis, showing that CD54 high expression group had more T4 stage than CD54 low expression group. But no clinicopathological features were independently associated with CD54 expression.

In qPCR, 53 (57.0%) and 40 (43.0%) were divided into CD54 low expression and CD54 high expression groups, but no clinicopathological characteristics were significantly related to CD54 expression.

### Sox2

In IHC, 307 patients were divided into Sox2 low (n=211, 68.7%) and high (96, 31.3%) expression groups. Sox2 high expression was significantly correlated with well or moderate differentiation grade both in univariate (p=0.042) and multivariate (p=0.041, OR=0.699, 95%CI [0.495-0.986]) analyses.

In qPCR, 43 (46.2%) and 50 (53.8%) patients were divided into Sox2 low expression and high expression groups. Sox2 expression was notably associated with age ≤60 years (p=0.008) and T3-4 stage (p=0.030) in univariate analysis. In logistic regression, only age was the independent related factor to Sox2 (p=0.009, OR=0.322, 95%CI [0.137-0.755]).

**Figure 3:**
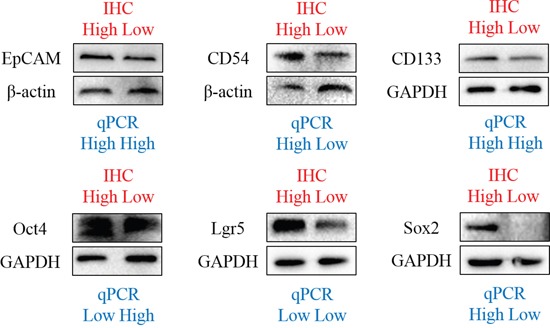
Western blot of markers in some patients

In primary lesions (n=93) tested by qPCR and IHC, we found that there were no relationships between IHC and qPCR in CD54 (p=0.477), Lgr5 (p=0.576), CD133 (p=0.792), Oct4 (p=0.834), EpCAM (p=0.630) and Sox2 (p=0.250). We have also compared the expression of these markers in some patients through Western Blot, the results of which was similar to IHC, but not consistent with qPCR in EpCAM, CD133, Oct4 and Lgr5 (Figure 3).

### Prognostic significance of the expressions of these markers in primary lesions

In this study, 341(90.5%) patients in IHC and 89 (95.7%) in qPCR were followed up. But we only included 325 (86.2%) patients in IHC (Sox2: 86.0%, 264/307) and 80 (86.0%) patients in qPCR with R0 resection to perform the survival analyses. The median survival time (MST) and 2-year overall survival rates of different groups of these markers in IHC and qPCR were shown in Table 5. The MST was not applicable when the survival rates at the end of follow-up time were still higher than 50%.

**Table 5:** The median survival time and 2-year overall survival rates of the patients with R0 resection in survival analyses in this study

Markers	Groups	Patients in IHC	Immunohistochemistry		Patients in qPCR	Quantitative PCR	
		Follow-up rate (%)	Median survival time (months)	2-year overall survival rate	Follow-up rate (%)	Median survival time (months)	2-year overall survival rate
	All	325/377, 86.2%	65.4	75.1%	80/93, 86.0%	—	68.8%
Lgr5	Low	233/276, 84.4%	68.2	77.2%	45/50, 90.0%	30.4	60.0%
	High	93/101, 92.1%	55.3	70.0%	35/43, 81.4%	—	80.0%
Oct4	Low	235/266, 88.3%	69.7	78.3%	38/44, 86.4%	32.6	65.8%
	High	90/111, 81.1%	54.1	66.7%	42/49, 85.7%	—	71.4%
CD133	Low	271/311, 87.1%	68.2	75.3%	59/69, 85.5%	—	78.0%
	High	54/66, 81.8%	46.6	74.1%	21/24,87.5%	18.0	42.9%
EpCAM	Low	109/123, 88.6%	—	80.7%	55/65,84.6%	—	74.5%
	High	216/254, 85.0%	54.5	72.2%	25/28, 89.3%	29.1	56.0%
CD54	Low	281/321, 87.5%	68.0	75.8%	45/53,84.9%	—	73.3%
	High	44/56, 78.6%	36.0	74.5%	35/40, 87.5%	32.3	62.9%
Sox2	Low	180/211, 85.3%	59.0	71.1%	38/43, 88.4%	32.8	63.2%
	High	84/96, 87.5%	—	79.8%	42/50, 84.0%	—	73.8%

**Figure 4:**
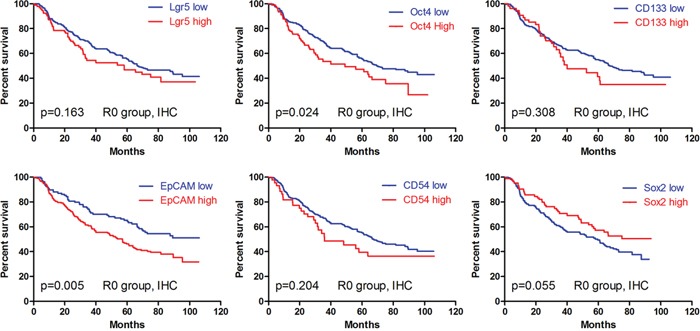
Kaplan-Meier analyses of these markers in IHC

**Figure 5:**
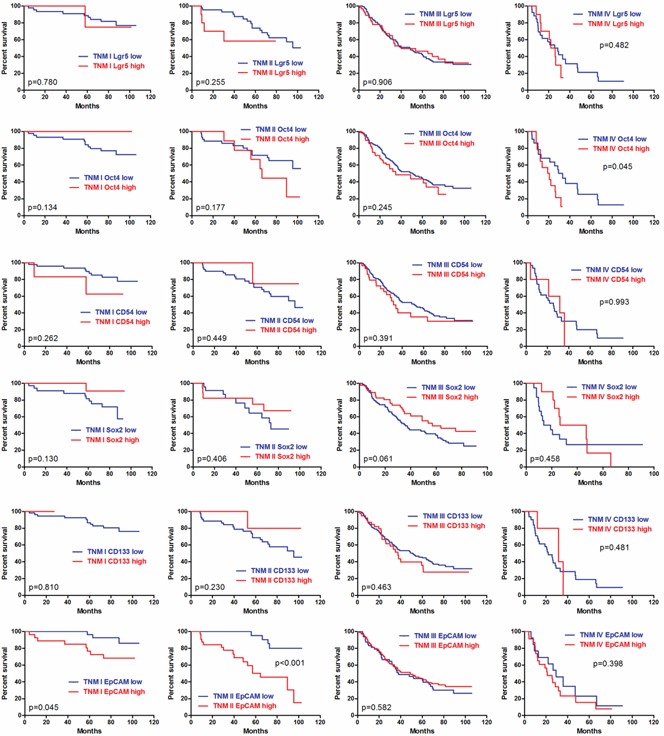
Prognosis stratified by TNM stage in IHC

**Figure 6:**
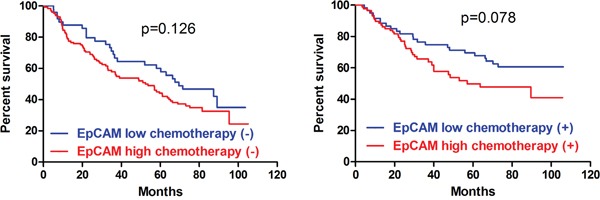
Prognosis stratified by chemotherapy of EpCAM

In IHC, we found that the patients with Oct4 (p=0.024) and EpCAM (p=0.005) high expressions had significantly worse prognosis than those with low expression in Kaplan-Meier analyses (Figure 4). The differences between low expression and high expression of Lgr5 (p=0.163), CD133 (p=0.308), CD54 (p=0.204) and Sox2 (p=0.055) were not significant (Figure 4). To eliminate the potential bias from TNM stage, we compared the prognosis between low and high expressions of these markers stratified by TNM stage (Figure 5). We found that the prognostic differences were significant between Oct4 low and high expressions in TNM IV stage (p=0.045), EpCAM low and high expression in TNM I stage (p=0.045) and TNM II stage (p<0.001). We found that more patients received chemotherapy in EpCAM low expression group than EpCAM high expression group (p=0.003). To eliminate the influence of chemotherapy, we compared the prognosis of patients with or without chemotherapy between EpCAM low and high expression. Kaplan Meier curve showed that although there were no significant differences between EpCAM low and high expression in patients with (p=0.078) or without chemotherapy (p=0.126), the trend that EpCAM high expression group had worse prognosis than low expression group was still visible (Figure 6). In Cox regression of patients with all markers tested except Sox2, no markers were the independent prognostic factors. Instead, we found that age (p=0.006, HR=1.303, 95%CI [1.052-1.613]), tumor size (p=0.041, HR=1.255, 95%CI [1.010-1.559]) and TNM stage (p<0.001, HR=2.038, 95%CI [1.633-2.544]) were independently related to prognosis. For Cox regression of patients with Sox2 tested, we found that age (p=0.001, HR=1.477, 95%CI [1.165-1.873]), Sox2 (p=0.013, HR=0.616, 95%CI [0.421-0.901]) and TNM stage (p<0.001, HR=2.008, 95%CI [1.597-2.526]) were independently associated with prognosis. The results showed that the patients with Sox2 high expression had significantly better survival outcomes than those with Sox2 low expression.

**Figure 7:**
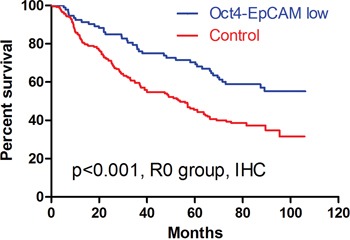
Kaplan-Meier analyses of Oct4-EpCAM in IHC

Because Oct4 and EpCAM were significantly related to prognosis in univariate analyses, we combined Oct4 and EpCAM to investigate their prognostic significance. The patients with both Oct4 and EpCAM low expressions were subsequently enrolled into double low expressions group (26.8%, 101/377) and other patients were in control group (73.2%, 276/377). Finally, out of 325 patients in survival analyses, 93 (28.6%) patients in Oct4-EpCAM double low expression group had significantly better prognosis than 232 (71.4%) patients in control group in Kaplan-Meier analysis (p<0.001) (Figure 7). Multivariate survival analyses indicated that age (p=0.036, HR=1.259, 95%CI [1.016-1.561]), Oct4-EpCAM expression (p=0.035, HR=1.485, 95%CI [1.028-2.145]) and TNM stage (p<0.001, HR=2.133, 95%CI [1.720-2.646]) were independent prognostic factors in IHC.

**Figure 8:**
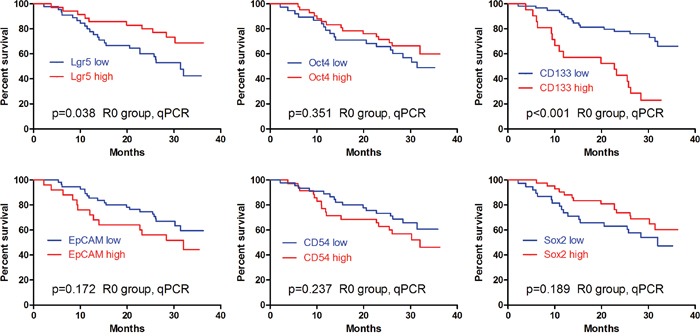
Kaplan-Meier analyses of these markers in qPCR

In qPCR, the univariate survival analyses revealed that the patients with Lgr5 low expression (p=0.038) and CD133 high expression (p<0.001) had significantly worse prognosis than those with Lgr5 high expression and CD133 low expression, respectively (Figure 8). Contrarily, no significant differences in prognosis were found between the low and high expression of Oct4 (p=0.351), CD54 (p=0.237), EpCAM (P=0.172) and Sox2 (p=0.189) (Figure 8). In multivariate analyses, only CD133 was demonstrated to be independently related to survival outcomes (p<0.001, HR=4.338, 95%CI [2.152-8.747]).

### Nomogram of prognostic prediction based on Oct4-EpCAM expression in primary lesions in IHC

**Figure 9:**
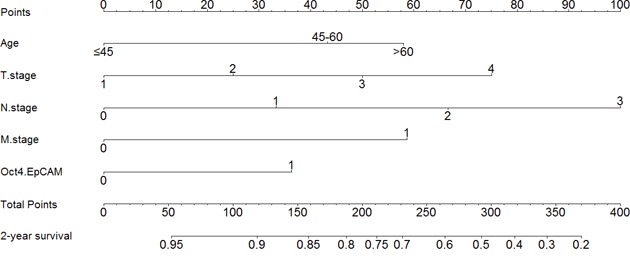
Nomogram based on Oct4-EpCAM

**Figure 10:**
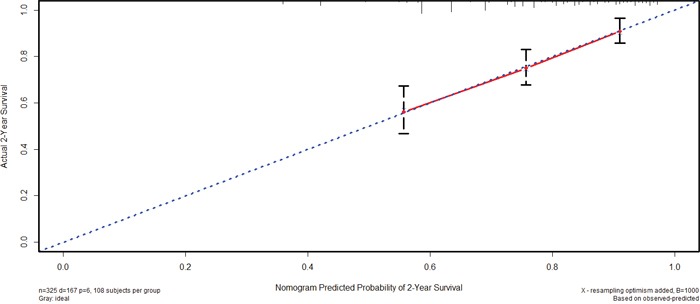
Calibration of nomogram based on Oct4-EpCAM

**Figure 11:**
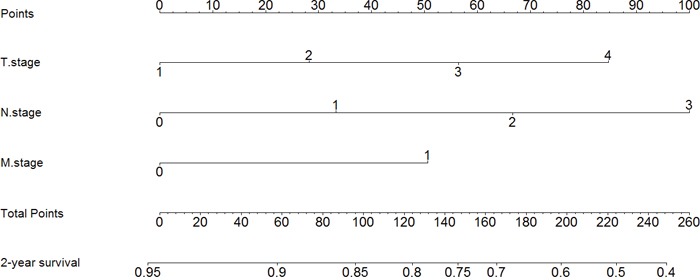
Nomogram based on TNM stage alone

**Figure 12:**
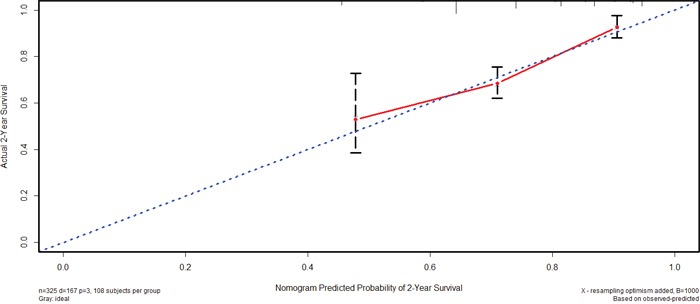
Calibration of nomogram based on TNM stage alone

Further, we used nomogram to predict 2-year overall survival rate of individual patient in IHC. The results showed that age, T stage, N stage, M stage, and Oct4-EpCAM expression (p=0.040, HR=1.484, 95%CI 1.019-2.160) were included in the nomogram (Figure 9), indicating that Oct4-EpCAM double low expression group had better survival outcomes, which was similar to that of aforementioned multivariate analyses. The calibration curve of nomogram showed that the predictive probability of 2-year survival were very closely to the actual 2-year survival (Figure 10). Subsequently, we compared the predictive accuracy of prognosis between the nomograms based on Oct4-EpCAM expression and TNM staging (only T stage, N stage and M stage included, Figure 11, 12). The C-indexes of nomograms were 0.711 (95%CI 0.676-0.746), compared with 0.698 (95%CI 0.659-0.737) of TNM staging system in this study. The results indicated that the prognostic prediction accuracy of nomograms based on Oct4-EpCAM expression and other parameters was significantly better than TNM staging system alone (p=0.003).

### Clinical significance of these markers in metastatic LNs in IHC

In 275 patients with positive N stage, we collected 206 (74.9%) metastatic LNs to perform IHC. There were 24 (11.7%) patients in CD54, 45 (21.8%) in Lgr5, 23 (11.2%) in CD133, 43 (20.9%) in Oct4 and 167 (81.1%) in EpCAM in high expression groups. For Sox2, out of 223 patients with positive N stage, 185 (83.0%) metastatic LNs were collected to be investigated by IHC, and the high expression rate was 33.5% (n=62). All the expressions of these markers in metastatic LNs had significantly relation to the expressions in primary lesions (all p<0.001).

EpCAM high expression was significantly associated with old age (p=0.011), greater curvature and anterior wall of stomach (p=0.034), larger tumor size (p=0.036). Multivariate analysis showed that only age was independently related to EpCAM expression (p=0.004, OR=2.031, 95%CI [1.259, 3.277]). Oct4 high expression was related to N3 stage (univariate analysis: p=0.044, multivariate analysis: p=0.042, OR=1.812, 95%CI [1.021, 3.215]). CD133 high expression was correlated with well or moderate differentiation (p<0.001). Multivariate analysis also showed that longitudinal location (p=0.043, OR=0.571, 95%CI [0.332, 0.982]) and differentiation (p<0.001, OR=0.299, 95%CI [0.165, 0.541]) were independent related factors to CD133 expression. In univariate analyses, Lgr5 and CD54 low expression were significantly connected to middle part of stomach (p=0.022) and female (p=0.004), respectively. Sox2 low expression were significantly related to old age (univariate analysis: p=0.007, multivariate analysis: p=0.016, OR=0.594, 95%CI [0.389, 0.907]).

In Kaplan-Meier analyses, there were no significant differences between high expression and low expression groups of CD54 (p=0.610), Lgr5 (p=0.053), Sox2 (p=0.858), EpCAM (p=0.733) and CD133 (P=0.276). However, Oct4 high expression group had significantly worse prognosis than Oct4 low expression group (p=0.006), and we found that Oct4 was also an independent prognostic factor in Cox regression (p=0.034, HR=1.626, 95%CI [1.038-2.547]).

## DISCUSSION

The accurate prognostic prediction of GC are pivotal in clinical practice. At present, as the main method, TNM stage system is widely applied to reveal tumor progression and predict prognosis of patients. Nevertheless, TNM stage can only reflect the general information of tumor progression. Because of the heterogeneity, the individual specific characteristics of tumors and patients cannot be completely revealed only through TNM stage system. With the development of biological technology, the heterogeneity of genetics, like proteomics, genomics, has been gradually discovered in GC [12]. Therefore, besides TNM stage, it is very important to take the heterogeneity of GC into consideration in prognostic prediction. Additionally, CSCs, as the putative source of tumor maintaining and therapy resistance, have also been investigated deeply and widely in many tumors in recent years. In this present study, we focused on the expressions of specific markers of CSCs in GC tissues to find out their clinical significance in GC and potential application in clinical practice.

In this study, we investigated the expressions of Lgr5, Oct4, CD133, EpCAM, CD54 and Sox2 through IHC and qPCR in GC tissues. In IHC, multivariate analyses demonstrated that Lgr5, CD133 and EpCAM were independently related to old age, CD133 and Sox2 were independently associated with well or moderate differentiation, while Oct4, CD133 and EpCAM were independently related to tumor progression. Regarding qPCR, logistic regression analyses showed that Lgr5 and Sox2 were independently related to well or moderate differentiation and young age, respectively. With respect to prognosis in IHC, we only found that the patients with high expression of Oct4 and EpCAM had significantly worse survival outcomes than those with low expression in univariate analyses. The differences between the high and low expression groups of other four markers were not significant. However, none of these six markers were independent prognostic factors. Based on the differences in survival outcomes between the patients in high and low expression of Oct4 and EpCAM, we combined Oct4 and EpCAM together to investigate the survival outcomes between low expression of Oct4-EpCAM and high expression of Oct4/EpCAM (control group). Cox regression showed that Oct4-EpCAM was the independent prognostic factor. In qPCR, we only found that CD133 was an independent prognostic factor, indicating that the patients with CD133 high expression had significantly worse prognosis than those with CD133 low expression.

Lgr5 was identified as the putative marker of CSCs in colon cancers [13]. Lgr5 was found to be related to depth of invasion, LNs metastasis, distance of metastasis and poor prognosis, and after Lgr5 was inhibited by siRNAs, fewer GC cells migrated through transwell model [14]. Lgr5 had also been considered as an important marker in carcinogenesis of GC, indicating that Lgr5 expression was gradually increased from normal control tissues to GC tissues [15]. Lgr5 was also been studied as a potential novel biomarker in chemoresistance of GC cells and predicting response to chemotherapy and prognosis [16]. However, other study demonstrated that Lgr5 was increased expressed in well-moderate differentiation, stage I and stage II, compared with stage III and stage IV [17].

Oct4 was identified as the putative marker of oral cancer stem-like cells and played a pivotal role in the chemoresistance of CSCs derived from prostate cancer [18, 19]. Previous study showed that GC patients with negative expression of Oct4 had worse prognosis than those with positive expression [20]; however, other report showed that Oct4 was expressed higher in GC tissues than non-cancerous tissues and associated with poor differentiation [21]. It was demonstrated that metastatic lesions had more Oct4 positive expression than negative expression [22].

CD133 has been widely investigated as the specific marker of CSCs of brain tumors, prostate cancer, melanoma and pancreatic cancer, but there are still some controversial results indicating that CD133 negative cells might also include CSCs [4, 5, 23, 24]. In our study, CD133 was generally expressed in the lumen of carcinoma glands, which was also reported by other previous research [25]. However, besides luminal expression, cytoplasmic location was another kind of expression and previous research showed that the cytoplasmic expression of CD133 was related to metastasis and tumor progression, but this relationship was not observed in luminal expression [25]. The GC patients with CD133 positive expression were related to poorly differentiation, and had significantly poor survival outcomes than those with CD133 negative expression [26–27]. In our study, although CD133 expression was related to well or moderate differentiation, CD133 expression was also associated with N2-3 stage, which was also similar to previous studies [28–29]. However, we did not found that CD133 was related to survival outcomes in IHC. Instead, we demonstrated that the patients with CD133 high expression had significantly worse prognosis through qPCR.

EpCAM has also been targeted as the putative marker of epithelial CSCs of ovarian cancer and pancreatic cancer [24, 30]. EpCAM was found significantly related to large tumor size and poor survival outcomes through IHC, which was similar to a previous study [31]. EpCAM was also found high expressed in peritoneal metastasis of GC, indicating that only GC cells with high expression of EpCAM might metastasize to the peritoneum [32]. In some experiments, the capabilities of cell proliferation and tumor formation in nude mice of GC cell lines were impaired after EpCAM downregulation [33]. However, another study reported that the patients with loss of EpCAM expression had significantly worse prognosis than those without loss and in stage I and II disease, loss of EpCAM expression was related to aggressive tumors [34].

CD54 was found as the surface marker of cancer stem cells of hepatocellular carcinoma, GC and rectal cancer [6, 35, 36]. We found that CD54 was not independently associated with any clinical pathological characteristics and CD54 was not related to prognosis. However, a previous report with 108 patients demonstrated that CD54 was significantly related to advanced stage and liver metastasis [37]. Additionally, many reports found that serum level of soluble CD54 was closely associated with GC progression, hematogenous metastasis and prognosis [7, 8].

Sox2 was expressed in the spheres of glioblastoma and gliosarcoma, and also played an important role in the epithelial mesenchymal transition of glioma stem cells [38]. Our study found that Sox2 was expressed in nuclei and cytoplasm. Some studies found that Sox2 high expression might be associated with invasion of gastric cancer and poor survival outcomes [20, 22, 39]. Loss of expression of Sox2 indicated a worse prognosis [40]. However, we found that Sox2 high expression was a favorable prognostic factor.

In this study, we used X-tile software to calculate the cut-point of each marker tested through IHC and qPCR to divide the patients into low expression and high expression groups. These cut-points were on the basis of survival data. Hence, we thought that the cut-points could reveal the differences between low and high expression more realistically. Additionally, we used monoclonal antibodies of all these markers in IHC to try our best to make the results more specific and solid. However, polyclonal antibodies were used in some studies [25, 17, 21, 29, 37, 41–44]. We thought that different types of antibodies might also be the reason of the variable high expression rates among our study and some previous ones. Moreover, we applied C-index to compare the accuracy of prognostic prediction and nomogram, a visualized method based on several valuable parameters to illustrate the prognosis of individual patients in this study. Through C-index and nomogram, we found that the prognostic prediction of nomogram based on Oct4 and EpCAM, age and TNM stage had significantly better accuracy than TNM alone, which indicated that the expressions of Oct4 and EpCAM were valuable in prognosis prediction of patient with GC. The results suggested that we should not only focus on TNM stage, but also pay attention to some specific characteristics of patients and tumors. However, we found that few previous studies had applied these kinds of methods in GC.

Our study applied IHC and qPCR to investigate the protein and mRNA expression of GC tissues. Moreover, qPCR also tested the mRNA expression of normal gastric tissues. These might be the reason why the results of IHC and qPCR were not correlated with each other. The results of Western Blot was similar to IHC, but not consistent with qPCR in some markers. We thought that the expression of mRNA in qPCR might differ from the expression of protein in IHC because of the changes after transcription and transduction. Additionally, our study only investigated the clinical significance of these markers, but did not involve the molecular mechanism of these markers. At present, these markers were mainly applied in identification of CSCs and investigation of their clinical significance. Regarding the mechanism, some study found that TR4-Oct4-IL1Ra axis might play a critical role in the development of chemoresistance in the prostate cancer stem/progenitor cells [45]. GC cell migration was enhanced through increasing CD54 through Rho/ROCK pathway by leptin [46].

There were still some limitations in our study. This study was a retrospective one with 377 patients in IHC and 93 patients in qPCR. Because of the difficulty and feasibility of GC tissues, we could only collect and test GC tissues with enough size to avoid the influence of postoperative pathological examination. Therefore, most GC tissues in qPCR were TNM III-IV stage. In addition, CD44 was another very important marker of GC and gastric CSCs and had been reported widely. In this study, we mainly focused on these six markers with fewer reports. Additionally, this study only enrolled the patients in our hospital, and the results should be still further demonstrated through external validation.

In conclusion, our study showed that low expressions of Oct4-EpCAM in IHC and CD133 in qPCR were favorable prognostic factors in GC. The nomogram based on the expression of Oct4-EpCAM was accurate and valuable in prognostic prediction of patients with GC.

## MATERIALS AND METHODS

### Patients

**Figure 13:**
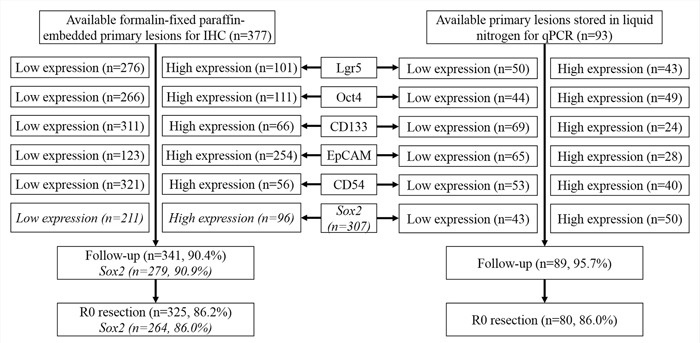
Flow chart of the patients in this study

Available formalin-fixed paraffin-embedded primary lesions (n=377) and metastatic LNs (n=194) of patients with GC in Department of Gastrointestinal Surgery, West China Hospital, Sichuan University from January 2006 to October 2012 were retrospectively enrolled for CD54, Lgr5, Oct4, CD133 and EpCAM tests through IHC. For Sox2, available formalin-fixed paraffin-embedded primary lesions (n=307) and metastatic LNs (n=184) were collected from January 2007 to October 2012. We also collected 93 pairs of primary lesions and corresponding adjacent normal gastric tissues stored in liquid nitrogen of patients with GC from December 2011 to October 2012 to perform qPCR. The primary lesions of these 93 patients had also been tested by IHC. All patients were followed up through telephones, mails and outpatient visits up to January 2015. The clinicopathological characteristics and follow-up details were collected. The West China Hospital research ethics committee approved retrospective analysis of anonymous data. Signed patient informed consent was waived per the committee approval, because it was a retrospective analysis. The flow chart of the patients was shown in Figure 13.

### IHC of GC tissues and immunofluorescence of GCSCs spheres

The tissue slices (4 μm) were deparaffinized with xylene and rehydrated in a graded alcohol series and distilled water. After blocking the endogenous peroxidase with hydrogen peroxide, citrate buffer (ZhongShan Golden Bridge Biotechnology Co., Ltd) was used to perform antigen retrieval in water bath at 95°C for 35 minutes. After naturally cooling down, the slices were incubated with primary monoclonal antibodies to CD54 (1:75, Abcam), Lgr5 (1:60, Abcam), Oct4 (1:200, Abcam), EpCAM (1:800, Abcam), Sox2 (1:200, ProMab) at 4°C and with primary monoclonal antibody to CD133 (1:10, Miltenyi Biotec) at room temperature overnight. For immunofluorescence, the frozen slices (8 μm) of GCSCs spheres were incubated with these primary monoclonal antibodies under the same conditions, except EpCAM (1:200, Abcam). Subsequently, these slices were incubated with peroxidase-conjugated polymer (EnVision™ Detection Kit, Gene Tech (Shanghai) Company Limited) for 30 minutes or fluorescent secondary antibody (1:800, Alexa Fluor 555) for 90 minutes at room temperature. Finally, the slices were stained with diaminobenzidine chromogen solution (1:50, EnVision™ Detection Kit, Gene Tech (Shanghai) Company Limited) and counterstained with hematoxylin (ZhongShan Golden Bridge Biotechnology Co., Ltd) or DAPI (1:4000). Primary antibody incubation was omitted in negative controls. The specificity of all antibodies was demonstrated by providers. The figures were captured through Axio Imager A2 (Zeiss) and Scope A1 (Zeiss). As reported previously, GCSCs were cultured in serum free DMEM/F12 medium supplemented with 20 ng/ml EGF and 10 ng/ml b-FGF [6].

### qPCR

Total RNA was isolated from GC tissues by TRIzol (Invitrogen) according to the instructions. Reverse transcription of total RNA was carried out with PrimeScript RT reagent kit (TAKARA Biotechnology (Dalian) Co., Ltd) on PCR amplifier under the following conditions: 37°C for 15 min, 85°C for 5 seconds. After that, cDNA was tested in real-time qPCR on the CFX96 Real Time PCR System with the use of Premix Ex Taq (SYBR and probe qPCR, TAKARA Biotechnology (Dalian) Co., Ltd) under the following conditions: 95°C activation for 30 seconds, 95°C denaturation for 5 seconds, 60°C annealing and elongation for 30 seconds, which repeated for 40 cycles. The results were recorded with CT value. After comparison of amplification efficiency, Livak method (2^−ΔΔCT^) was used to compare the difference between GC tissues and corresponding adjacent normal gastric tissues, in which GAPDH was used as reference gene and corresponding adjacent normal gastric tissues were applied as calibration control. All the sequences of primers and probes of these genes were designed and synthesized by TAKARA Biotechnology (Dalian) Co., Ltd (Table 6).

**Table 6:** The sequences of primers and probes in quantitative in this study

PCR	Genes	Primers/probes	Sequences	Products
Probe	GAPDH	Forward	5′-GGACCTGACCTGCCGTCTAG-3′	98bp
		Reverse	5′-TAGCCCAGGATGCCCTTGAG-3′	
		Probe	5′-(FAM)CCTCCGACGCCTGCTTCACCACCT(Eclipse)-3′	
	Oct4	Forward	5′-GTGGGTAGGTTATTTCTAGA-3′	152bp
		Reverse	5′-GCAGAAGACTTGTAAGAAC-3′	
		Probe	5′-(FAM)AGGCAGAGGCACTTCTACAGAC(Eclipse)-3′	
	EpCAM	Forward	5′-GTCAGAAGAACAGACAAG-3′	180bp
		Reverse	5′-ACTCGTGATAAATTTTGGA-3′	
		Probe	5′-(FAM)CTCTGAGCGAGTGAGAACCTACT(Eclipse)-3′	
	CD133	Forward	5′-ACTCCAGAGCAAATCAAA-3′	91bp
		Reverse	5′-CTAGCACTGAATTGATACTG-3′	
		Probe	5′-(FAM) ACACTACCAAGGACAAGGCGT (Eclipse)-3′	
SYBR	GAPDH	Forward	5′-TCAACAGCGACACCCACTC-3′	106bp
		Reverse	5′-GCTGTAGCCAAATTCGTTGTC-3′	
	CD54	Forward	5′-GAGCCAATTTCTCGTGCCG-3′	108bp
		Reverse	5′-GTCGCTGGCAGGACAAAGGT-3′	
	Lgr5	Forward	5′-AATGCCTTATGCTTACCAGTGCT-3′	102bp
		Reverse	5′–AGGTCGTCCATACTGCTGTTGTC-3′	

### Western Blot

The protein of gastric cancer tissues was extracted through RIPA Buffer (Aidlab) with protease and phosphatase inhibitor cocktails (Roche). BCA protein assay (KeyGen biotech) was used to quantify protein concentration. The protein (30 μg) was separated in 10% Tris Glycine SDS gels and transferred to polyvinylidene difluoride membranes (Millipore). The membranes were blocked with 5% milk in TBST for 60 minutes at room temperature, then incubated with primary antibodies overnight at 4°C. After incubating with secondary antibodies (ZhongShan Golden Bridge Biotechnology Co., Ltd), the membranes were tested with Super Signal West Femto Masimun sensitivity substrate (Thermo Scientific).

### Definition of cut-points

In IHC, staining percentage scores (0-5 points) multiplied staining intense scores (0-5 points) to get IHC scores (0-25 points). Staining percentage scores were defined as 0 point (0%-5%), 1 point (6%-25%), 2 points (26%-50%), 3 points (51%-75%), 4 points (76%-95%) and 5 points (96%-100%). Staining intense scores were defined as 0 point (negative), 1 point (weak), 2 points (weak to moderate), 3 points (moderate), 4 points (moderate to strong) and 5 points (strong). With the use of X-tile software (Version 3.6.1, Yale University), the optimal cut-points for IHC were analyzed and calculated as 12 points of CD54, 0 point of Lgr5, 0 point of CD133, 0 point of Oct4, 6 points of EpCAM and 8 points of Sox2 in primary lesions. The cut-points of these markers in metastatic LNs were the same with primary lesions, except Lgr5 (5 points) and Oct4 (1 point). Regarding qPCR, the cut-points of the expression folds of GC tissues to corresponding adjacent normal gastric tissues were also analyzed and calculated through X-tile. The cut-points were 1.5 folds of CD54, 1.3 folds of Lgr5, 2.5 folds of CD133, 0.6 folds of Oct4, 7 folds of EpCAM and 0.2 folds of Sox2. According to the cut-points in IHC and qPCR, the patients were further divided into low expression groups (≤cut-points) and high expression groups (>cut-points).

### Statistical analyses

Statistical analyses were mainly conducted by SPSS software (Version 22, IBM). Chi-square test and rank sum test (Mann-Whitney U test) were used to analyze the unordered categorical variable and ranked data, respectively. Student's t-test was used to analyze the continuous data, if homogeneity of variance and normal distribution. Otherwise, rank sum test was used. Logistic regression was applied in multivariate correlation analysis. Kaplan-Meier and life-table methods were used to calculate the cumulative survival rates. Log-rank test and Cox's proportional hazard regression model were conducted for univariate and multivariate survival analyses, respectively. Prism 5 for Windows (Version 5.01, GraphPad Software) was used to draft the figure of Kaplan-Meier curve. Nomograms and calibration curves were performed through R for Windows (Version 3.2.0, R Foundation for Statistical Computing) with the package of Regression Modeling Strategies (rms), in which the variables were selected according to the model by Akaike information criterion in a stepwise algorithm. Comparison between the nomogram and TNM stage was performed with the package of Harrell Miscellaneous (Hmisc) and was evaluated by C-index meaning that the larger C-index, the more accurate was the prognostic prediction. Two-sided p value less than 0.05 was considered as statistical significance.
